# Causal Links Between Antibody-Mediated Immune Responses and Pancreatic Cancer Risk

**DOI:** 10.2174/0118715303396420251007140802

**Published:** 2025-11-03

**Authors:** Xinyun Zou, Yicheng Feng, Pei Liu, Ling Zhang, Jinlan Shen

**Affiliations:** 1 Department of Oncology, The General Hospital of Western Theater Command, Chengdu 610083, China;; 2 Department of Laboratory Medicine, The General Hospital of Western Theater Command, Chengdu 610083, China

**Keywords:** Antibody-mediated immune responses, pancreatic cancer (PC), mendelian randomization (MR), causal inference, sensitivity, immune activation, chronic inflammation

## Abstract

**Introduction:**

Pancreatic cancer (PC) presents unique challenges to traditional immunotherapy due to its immunosuppressive microenvironment and low mutation burden. Research into antibody-mediated immune responses, which has been extensively applied in various cancer types, is also applicable to the study of pancreatic cancer.

**Methods:**

Using public Genome-wide association study (GWAS) data and genetic instruments, we conducted a two-sample Mendelian randomization (MR) analysis to explore causal relationships between 46 antibody-mediated immune responses and PC. We employed five MR methods. Cochran’s Q statistic and corresponding p-values from the MR Egger and Inverse Variance Weighted methods assessed instrument heterogeneity. Egger intercept and MR pleiotropy residual sum and outlier (MR-PRESSO) methods detected potential horizontal pleiotropy, ensuring the robustness of our findings.

**Results:**

Our study identified causal links between five antibody-mediated immune responses and pancreatic cancer risk. *Chlamydia trachomatis* momp A antibody levels were inversely correlated, while Anti-*helicobacter pylori* IgG seropositivity, Polyomavirus 2 JC VP1 antibody levels, and Merkel cell polyomavirus VP1 antibody levels showed positive correlations. Notably, Anti-Merkel cell polyomavirus IgG seropositivity exhibited strong positive associations across Inverse variance weighting (IVW), weighted median, and MR-Egger analyses (*p* < 0.05).

**Discussion:**

These results suggest that specific Antibody-Mediated Immune Responses may modulate pancreatic carcinogenesis through immune activation or chronic inflammation. The protective association for *Chlamydia trachomatis* momp A contrasts with the risk-enhancing effects observed for anti-*helicobacter pylori* IgG and polyomaviruses, highlighting heterogeneous immunobiological pathways that warrant mechanistic investigation.

**Conclusion:**

Our study demonstrated a randomized causal effect between antibody-mediated immune responses and pancreatic cancer, offering a new perspective for clinical diagnosis and treatment of the disease.

## 1 INTRODUCTION

Pancreatic cancer (PC) is recognized globally as one of the most lethal malignancies, characterized by exceptionally high incidence and mortality rates, and notably poor survival outcomes. In 2023, it is estimated that approximately 66,440 new cases of PC will be diagnosed in the United States, with about 51,750 fatalities expected [1]. Globally, the year 2020 saw around 495,773 new diagnoses, underscoring the significant global burden of this devastating disease [2]. The intricacies of its origins, coupled with the difficulties in early detection, intensify its lethal nature [3, 4]. The development of immune tolerance in patients further complicates treatment, as it enables the cancer to avoid and diminish the immune response [5, 6]. Despite decades of research into these mechanisms, the pathways driving immune tolerance in pancreatic cancer remain poorly understood [7, 8]. This gap underscores the urgent need for continued investigation of pancreatic cancer’s immune dynamics to develop more effective therapies.

The antibody-mediated immune response, commonly referred to as humoral immunity, is primarily facilitated by B cells [9]. This response centers on antibodies—specialized proteins that B cells produce and release into the bloodstream and other bodily fluids [10]. As essential elements of the adaptive immune system, these responses play a vital role in fighting infections and creating long-term immune memory [11]. This memory allows for swift and robust reactions upon subsequent exposures to pathogens [12-14].Antibody-mediated immunity impacts tumor dynamics through various mechanisms, including antibody-dependent cellular cytotoxicity (ADCC), complement-dependent cytotoxicity (CDC), and the disruption of signaling pathways [15, 16]. Generally, this immune response inhibits and controls tumor growth. However, in situations such as antibody-dependent enhancement, pro-inflammatory signaling, or immune evasion, the response can paradoxically promote tumor growth and progression.

Research into antibody-mediated immune responses has been extensively applied across multiple cancer types. For example, in HER2-positive breast cancer, trastuzumab binds to and inhibits the overexpressed HER2 receptor, curbing tumor growth by disrupting its signaling pathways [17-19]. Similarly, Rituximab, which targets the CD20 antigen on B cells, promotes apoptosis, complement-dependent cytotoxicity (CDC), and antibody-dependent cellular cytotoxicity (ADCC) in lymphoma cells [20]. These examples underscore the therapeutic potential and versatility of antibody-mediated strategies in cancer treatment.

PC poses distinct challenges for traditional immunotherapy, primarily due to its immunosuppressive microenvironment and low mutation load [8]. Despite these challenges, antibody-mediated therapeutic strategies represent a promising research direction. Continued refinement of these approaches may yield more effective treatments for pancreatic cancer patients [21, 22]. By employing innovative immune-based strategies, researchers can address one of oncology's most challenging malignancies. Mendelian randomization (MR) uses genetic variants as natural experiments to infer causal relationships between modifiable exposures and health outcomes [23]. This instrumental-variable approach exploits the random genetic assortment during meiosis to reduce confounding inherent in observational epidemiology [24].

Two-sample MR has been used to explore causal relationships in various diseases. For example, my study [25] investigated the causal effect of protein level ratios on non-small cell lung cancer, while another study [26] assessed the relationship between gut microbiota and irritable bowel syndrome. Our study applies this method to investigate the causal link between antibody-mediated immune responses and the risk of pancreatic cancer.

## 2 MATERIALS AND METHODS

### 2.1 Research Design

This study's design and reporting adhere to the STROBE-MR (Strengthening the Reporting of Observational Studies in Epidemiology using Mendelian Randomization) guidelines.

In this study, we utilized MR to explore the causal link between antibody-mediated immune responses and PC, as depicted in Fig. (**1**). MR capitalizes on genetic variants, particularly single nucleotide polymorphisms (SNPs), as instrumental variables (IVs) [27, 28]. To validate our MR approach, the selected SNPs must meet three essential criteria: (1) there is a strong association between the SNPs and the exposure (antibody-mediated immune response); (2) the SNPs are not linked to any confounders of the exposure-outcome relationship; and (3) the SNPs affect the outcome (PC) solely through the exposure, without any alternative pathways [29, 30].

### 2.2 GWAS Data Sources for Pancreatic Cancer

The GWAS data for PC [31] were obtained from the 10th release of the FinnGen database, with details and download information provided in Table **1**. FinnGen's project [31] focuses on studying the genomes and national health register data of 500,000 individuals in Finland, with an emphasis on disease endpoints. This analysis includes data from 224,737 participants in FinnGen [31], covering 15 diseases previously examined in extensive GWAS. Additionally, a GWAS spanning 1,932 diseases identified 2,733 genome-wide significant associations (893 phenome-wide significant (PWS), *P* < 2.6 × 10^-11^) across 2,496 (771 PWS) independent loci involving 807 (247 PWS) endpoints [31].

### 2.3 Antibody-Mediated Immune Responses GWAS Data Sources

The GWAS data on antibody-mediated immune responses [32] were sourced from the UK Biobank. Detailed information and the download link are provided in Table **1**. The UK Biobank cohort provided up to 10,000 serological measurements of infectious diseases alongside genome-wide genotyping [33]. The study [32] utilized data from 13 pathogens to establish 46 phenotypes, including 15 seropositivity case-control phenotypes and 31 quantitative antibody measurement phenotypes. The analyses revealed a significant locus at the MHC for EBV (*P* = 1.9 × 10^-76^ to 3.1 × 10^-16^), JC virus (JCV; *P* = 1.7 × 10^-47^ to 4.5 × 10^-24^), and Merkel cell virus (MCV; *P* = 7.3 × 10^-37^ to 1.0 × 10^-18^), all showing robust associations [32]. As this research was a reanalysis of previously gathered and publicly available data, it did not necessitate additional ethical approval.

### 2.4 Selection of Instrumental Variables

We selected instrumental variables (IVs) with an association *p*<1.0×10^−5^, which is consistent with common practices in similar MR studies [25, 26]. To ensure IVs came from independent loci, we applied a linkage disequilibrium (LD) threshold of R^2^ < 0.001 and a clumping distance of 10,000 kb, utilizing the 1000 Genomes EUR data in the “TwoSampleMR” packages [34, 35]. After extracting key data such as the effect allele, effect size (including β-value, standard error, and *P*-value) for each SNP, we calculated the proportion of variation explained (R^2^) and the F-statistics to assess the strength of the instruments [34]. The equations used were R^2^ = 2 × MAF × (1 - MAF) × β^2^ and F = [R^2^ × (n - k - 1)] / [k × (1 - R^2^)], where “MAF” represents the minor allele frequency of the SNPs used as IVs, “n” is the sample size, and “k” is the number of IVs utilized [36].To reduce potential confounders associated with the SNPs, we used the PhenoScanner website (http://www.phenoscanner.medschl. cam.ac.uk/) and found no confounders linked to the SNPs [37]. The SNPs that have undergone rigorous statistical analysis and significance threshold screening are shown in Table **S1**.

### 2.5 Statistical Analysis

All analyses were conducted using R software version 4.3.0, available at http://www.Rproject.org. Our two-sample MR framework follows established pipelines that have been validated in proteome-wide and microbiome-wide settings [36]. We used the ‘TwoSampleMR’ package to assess the causal links between 46 antibody-mediated immune responses and PC. We applied five methods using the 'TwoSampleMR' package: inverse variance weighting (IVW), weighted median-based, simple mode-based, MR Egger, and weighted mode methods. Cochran’s Q statistic and corresponding *p*-values were utilized to evaluate heterogeneity among the selected instrumental variables (IVs) [28, 38]. To assess the heterogeneity of IVs, we used Cochran’s Q statistic [25, 39].To account for horizontal pleiotropy, the MR-Egger method was employed, indicating horizontal pleiotropy if the intercept term is statistically significant [40]. Additionally, the MR pleiotropy residual sum and outlier (MR-PRESSO) method within the same package was used to identify and exclude potential horizontal pleiotropic outliers that might influence the results significantly [41, 42]. Scatter plots and funnel plots were also generated; scatter plots confirmed that the findings were not impacted by outliers, while funnel plots underscored the robustness of the correlations and the absence of heterogeneity [43].

## 3 RESULTS

In this study, we rigorously examined the causal links between 46 antibody-mediated immune responses and PC utilizing five distinct analytical methods [28, 44]: MR-Egger, weighted median, IVW, simple mode, and weighted mode. The analysis results, as shown in Table **S2**, indicated that the F-statistics for these antibody-mediated immune responses range from 21.64 to 37.10, all exceeding 10 [28]. This suggested a reduced likelihood of encountering weak instrument bias.

We selected the IVW method, a mainstream statistical approach, for our analysis [45]. Based on its *p*-value of less than 0.05 [44], we found a causal relationship between five types of antibody-mediated immune responses and the risk of developing PC, as illustrated in Fig. (**2**). Notably, the levels of Chlamydia trachomatis momp A antibody were inversely correlated with the risk of PC (OR=0.949, 95% CI=0.902-0.999, *p*=0.04). The other four phenotypes were positively correlated with the risk of PC. According to the IVW results, these included Anti-*helicobacter pylori* IgG seropositivity (OR=1.120, 95% CI=1.008-1.244, *p*=0.04), Polyomavirus 2 JC VP1 antibody levels (OR=1.135, 95% CI=1.010-1.276, *p*=0.03), and Merkel cell polyomavirus VP1 antibody levels (OR=1.158, 95% CI=1.037-1.294, *p*=0.01).

For Anti-Merkel cell polyomavirus IgG seropositivity (OR=1.097, 95% CI=1.014-1.187, *p*=0.02), the results from inverse variance weighted, weighted median, and MR-Egger analyses all yielded *p*-values less than 0.05, indicating a strong positive correlation with the risk of PC. Additionally, the SNP scatter plot in Fig. (**3**) also demonstrated that all five analyses consistently revealed a positive correlation between these four antibody-mediated immune response phenotypes and the risk of PC.

We provided the raw data from our MR analysis, including the concordance data between exposure and outcomes (Table **S1**), along with detailed result data for each SNP (Table **S3**). We presented the stable results of the five antibody-mediated immune responses and their association with PC in Fig. (**S1-S5**).

To assess the stability of our findings, we employed Cochran's Q test, MR-Egger, and Inverse Variance Weighted methods to check the heterogeneity of the selected SNPs. The results, as depicted in Fig. (**2**) and Table **S2**, indicated no heterogeneity with p-values greater than 0.05. Given the susceptibility of the IVW method to the effects of valid instruments and potential pleiotropic influences, we conducted sensitivity analyses to evaluate the robustness of the association. The MR-Egger method was used to test for horizontal pleiotropy, and the absence of such effects was confirmed as all *p*-values exceeded 0.05. These sensitivity analyses underscored the stability of our results.

## 4 DISCUSSION

Our Mendelian randomization analysis reveals compelling evidence for causal relationships between specific antibody-mediated immune responses and pancreatic cancer risk. The study identified five significant immune phenotypes showing both protective (Chlamydia trachomatis MOMP A antibodies) and risk-enhancing associations (Anti-Helicobacter pylori IgG, JC/Merkel cell polyomavirus antibodies) with pancreatic carcinogenesis. These findings illuminate previously unrecognized immunological pathways in pancreatic cancer development while presenting paradoxical associations that warrant mechanistic investigation. The following discussion contextualizes these results within existing oncological literature, examines potential biological mechanisms underlying the observed associations, and explores their implications for both diagnostic biomarker development and therapeutic targeting in pancreatic cancer management.

Chlamydia trachomatis MOMP A antibody levels measure the concentration of antibodies targeting the Major Outer Membrane Protein (MOMP) of *Chlamydia trachomatis*, a prevalent sexually transmitted infection [46-48]. This pathogen is associated with a range of health issues, including reproductive complications and a potentially heightened risk of certain cancers. A significant study highlighted a notable correlation between antibodies to *Chlamydia trachomatis* and an increased risk of ovarian cancer [49, 50]. Currently, there are no reports linking *Chlamydia trachomatis* MOMP A antibody levels with PC. Our research indicated a negative correlation between *Chlamydia trachomatis* MOMP A antibody levels and the risk of PC, providing new potential therapeutic targets from a whole-genomic perspective.

Anti-*Helicobacter pylori* IgG seropositivity indicates the presence of IgG antibodies against *Helicobacter pylori* in the blood, signifying past or current infection with this bacterium. Individuals with high levels of *H. pylori* IgG are at an increased risk of developing gastric and biliary cancers [51-53]. One study [54] reported that patients with higher titers of serum anti-*H. pylori* IgG (Hp-IgG) tends to be younger and have a higher incidence of diffuse-type gastric cancer. Additionally, another article [51] suggested that *H. pylori* infection significantly raises the risk of biliary cancer. There is also a case study [55] that reports a positive correlation between *Helicobacter pylori* infection and PC, and a population-based study by Saleem and Bleibel [56] found that *H. pylori* infection might be associated with an increased risk of PC. However, whether *H. pylori* is a risk factor for PC remains unclear, and more research is needed to clarify their exact relationship. Our study suggested that increased levels of Anti-*Helicobacter pylori* IgG seropositivity enhanced the risk of PC, further substantiating a positive causal relationship between them.

Polyomavirus 2 JC VP1 antibody levels measure the antibodies targeting the VP1 protein of the JC virus, a human polyomavirus. VP1 is the virus's major capsid protein and is essential for its ability to infect cells [57]. It is particularly important in monitoring and diagnosing conditions such as Progressive Multifocal Leukoencephalopathy (PML), especially in patients undergoing immunosuppressive therapy [58]. Literature indicates that JC virus's early protein, large T antigen (LT-Ag), promotes cell entry into the DNA synthesis phase (S phase) by interacting with cell cycle regulatory proteins, potentially facilitating tumor development [59-62]. This protein can interact with well-known tumor suppressors like p53 and Rb; through these interactions, the JC virus may contribute to cellular transformation into tumor cells [63]. Studies have detected JC virus DNA and proteins in various human tumors, including neurogenic and non-neurogenic tumors, and chronic inflammation caused by JCV infection may promote the selection and expansion of tumor cells [63, 64]. Although the role of JC virus is not fully understood, its presence has been linked to the development of colorectal cancer [64]. Our research indicated that increased levels of *Polyomavirus* 2 JC VP1 antibody were associated with a heightened risk of PC. Currently, no public literature explicitly connects this antibody immune response with PC, providing a new perspective for potential prevention and treatment strategies.

Anti-Merkel cell polyomavirus IgG seropositivity refers to the presence of IgG antibodies against the Merkel cell polyomavirus (MCPyV) in the serum [65]. MCPyV is associated with Merkel cell carcinoma (MCC) and is known for integrating its DNA into the human genome [66], continuously expressing small T antigen (ST) and truncated large T antigen (LT) [67-69]. Research involving the expression of MCPyV's LT and ST antigens in mouse skin has observed increased epidermal proliferation, cellular proliferation, and spontaneous tumor formation [70]; notably, the interaction of MCPyV LT antigen with the retinoblastoma protein (pRb) plays a significant role in these processes [68]. Currently, no literature suggests that levels of Anti-Merkel cell polyomavirus IgG seropositivity are related to the development of PC. However, our studies indicated a positive correlation, offering a new perspective for PC research. Merkel cell polyomavirus (MCPyV) VP1 antibody levels refer to the concentration of antibodies against the MCPyV VP1 protein in the blood. VP1, the primary capsid protein of MCPyV, is crucial for the virus’s ability to infect host cells [66]. The presence of these antibodies may indicate past or present infection with MCPyV [71]. Our research showed that elevated levels of this antibody were also associated with an increased risk of PC.

The elevated anti-Chlamydia trachomatis MOMP, A antibody titers, which have been found to have an inverse association with PC risk, may be leveraged to develop preventive vaccines or adjuvant therapies designed to enhance this protective immune response. In contrast, the positive correlations between anti-*Helicobacter pylori* IgG, Polyomavirus 2 JC VP1, and Merkel cell polyomavirus VP1 antibodies with PC risk indicated that these serologic markers could function as early indicators for individuals at high risk. This could guide more focused surveillance or personalized immunomodulatory treatments. The integration of these antibody profiles into patient stratification strategies for immune checkpoint blockade or vaccine trials holds significant potential, offering a pathway toward precision medicine in PC immunotherapy.

## CONCLUSION

This study provides a comprehensive MR analysis of 46 antibody-mediated immune responses and their causal effects on PC. Five immune phenotypes were significantly associated with PC incidence and progression, marking the first report of their link to PC, despite previous associations with ovarian, gastric, and skin cancers. These findings deepen our understanding of PC’s unique immune-related etiology and suggest novel therapeutic targets. By highlighting specific immune responses, this research supports the development of tailored immunotherapy strategies for PC, paving the way for improved prevention and treatment approaches.

## Figures and Tables

**Fig. (1):**
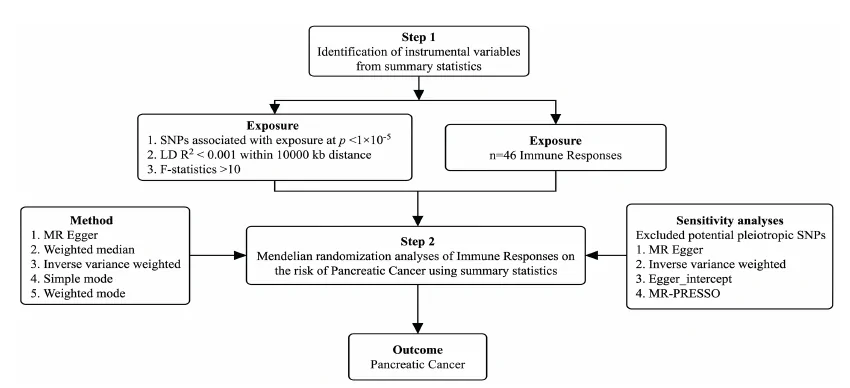
The study design of the associations of Antibody-Mediated Immune Responses and PC. **Abbreviations:** PC, pancreatic cancer, MR, Mendelian randomization; SNP, single nucleotide polymorphism.

**Fig. (2):**
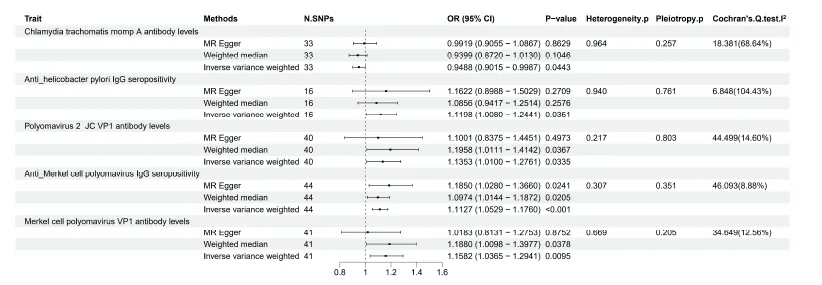
Forest plots showed the exploration of the causal effect of Antibody-Mediated Immune Responses onset on PC by using MR-Egger, weighted median, and IVW; **Abbreviations:** PC, pancreatic cancer; IVW: inverse variance weighting; CI: confidence interval.

**Fig. (3):**
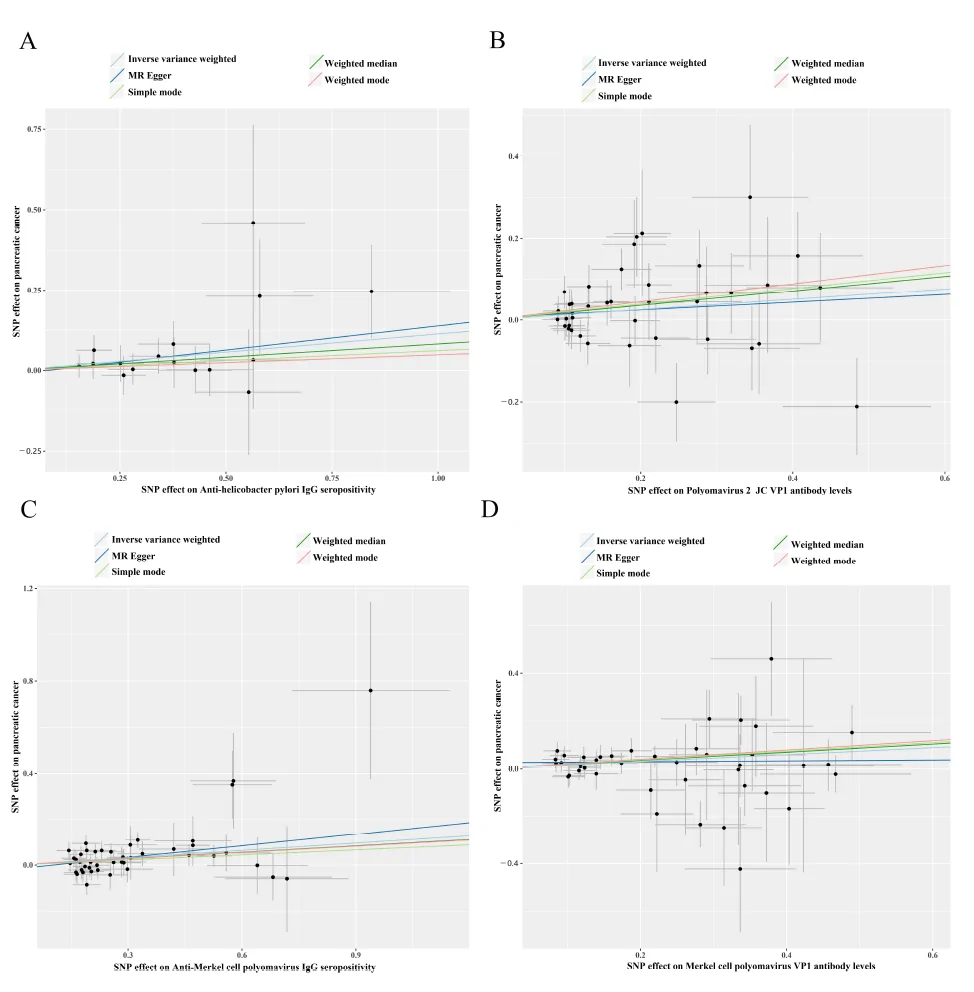
Scatter plot of the associations of genetic variants with four Antibody-Mediated Immune Responses and the risk of PC. **Abbreviations:** PC, pancreatic cancer; MR, mendelian randomization; SNP, single nucleotide polymorphism.

**Table 1:** Details of the genome-wide association studies and datasets used in our analyses.

**Exposure or Outcome**	**Sample Size**	**Ancestry**	**Links for Data Download**	**PMID**
Pancreatic Cancer	314193 controls and 1626 PC cases	FinnGen Project	https://storage.googleapis.com/finngen-public-data-r10/summary_stats/finngen_R10_C3_PANCREAS_EXALLC.gz	36653562
Antibody-Mediated Immune Responses	46 Phenotypes	UK Biobank	http://ftp.ebi.ac.uk/pub/databases/gwas/summary_statistics/(GCST90006884-GCST90006929)	33204752

## Data Availability

The data from this study, as well as the GWAS data of the subjects involved, are available from the corresponding author upon reasonable request. Links and supplementary materials provided in the article also offer access to our data, all of which are publicly accessible.

## LIST OF ABBREVIATIONS

PC: Pancreatic Cancer
MR: Mendelian Randomization
GWAS: Genome-Wide Association Study
CI: Confidence Interval
IVW: Inverse Variance Weighting
MR-PRESSO: MR Pleiotropy Residual Sum And Outlier
OR: Odds Ratio
SNPs: Single Nucleotide Polymorphisms
ADCC: Antibody-Dependent Cellular Cytotoxicity
CDC: Complement-Dependent Cytotoxicity
MOMP: Major Outer Membrane Protein
PML: Progressive Multifocal Leukoencephalopathy
MCPyV: Merkel Cell Polyomavirus
MCC: Merkel Cell Carcinoma

## STANDARDS OF REPORTING

STROBE guidelines were followed.
