# Supplementary material for: Causal Links Between Antibody-Mediated Immune Responses and Pancreatic Cancer Risk
Source: Endocr Metab Immune Disord Drug Targets. 2025 Nov 3;26:E18715303396420. doi: 10.2174/0118715303396420251007140802 (PMC13662728; doi:10.2174/0118715303396420251007140802)
Supplement: Supplementary file 1 [file EMIDDT-26-E18715303396420_SD1.pdf]

Supplementary Material

Causal Links Between Antibody-Mediated Immune Responses and Pancreatic Cancer Risk

Xinyun Zou[1], Yicheng Feng[1], Pei Liu[1], Ling Zhang[1] and Jinlan Shen[2,*]

[1]Department of Oncology, The General Hospital of Western Theater Command, Chengdu 610083, China; [2]Department of Laboratory Medicine, The General Hospital of Western Theater Command, Chengdu 610083, China

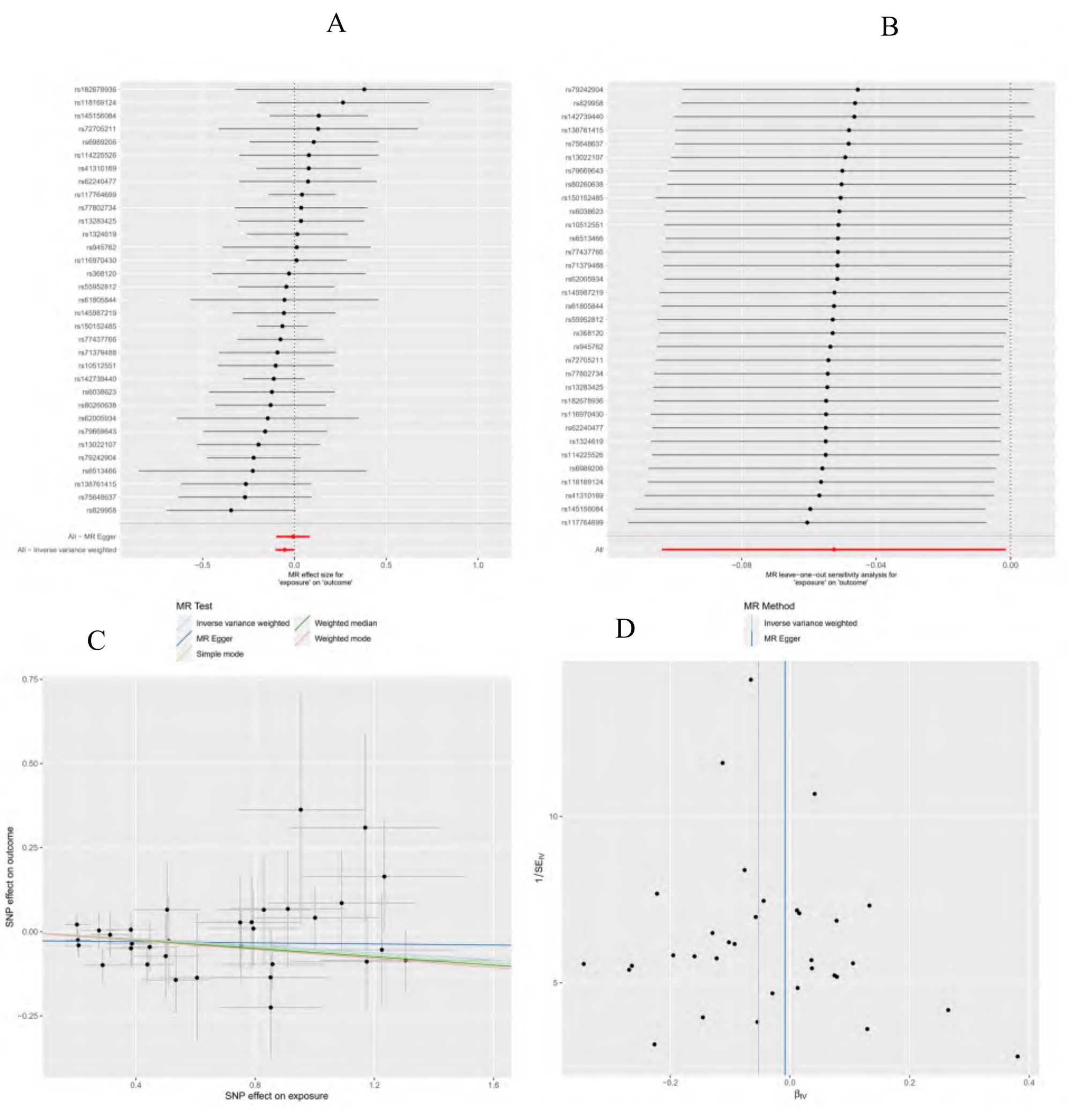

Fig. (S1): The stability of Chlamydia trachomatis momp A antibody levels was demonstrated by forest plot, scatter plot and funnel plot. (Causal effect of Chlamydia trachomatis momp A antibody levels on PC analysed by MR)
(A) Forest plot of the causal effects of single nucleotide polymorphisms associated with Antibody-Mediated Immune Responses on PC. (B) Leave-one-out of SNPs associated with Chlamydia trachomatis momp A antibody levels and their risk of PC. Each black point represents result of the IVW MR method applied to estimate the causal effect of Chlamydia trachomatis momp A antibody levels on PC excluding particular SNP. (C) Scatter plots of genetic associations with Chlamydia trachomatis momp A antibody levels against the genetic associations with PC. (D) Funnel plot to assess heterogeneity. PC = Pancreatic Cancer, SNP = single nucleotide polymorphism.

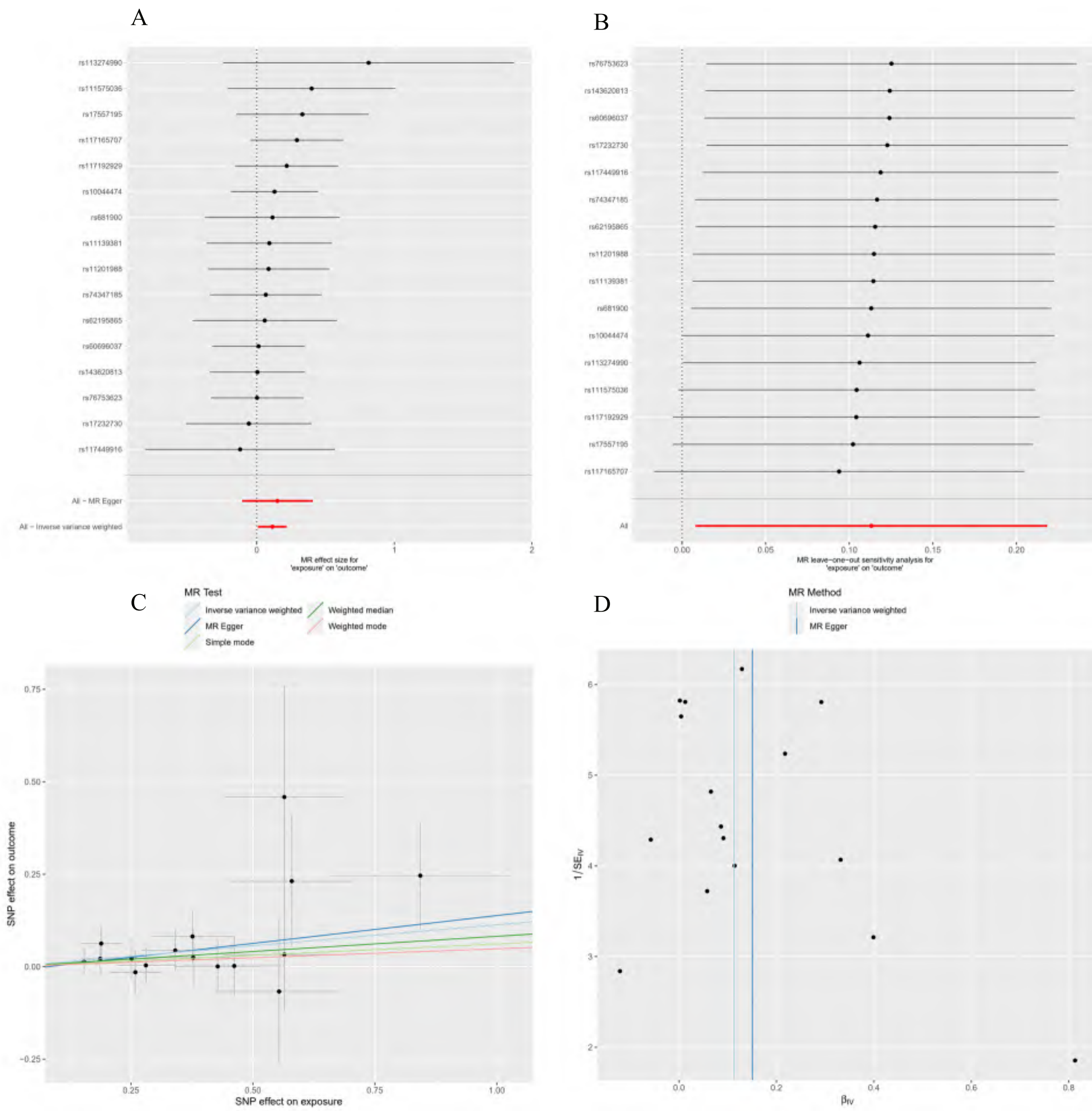

Fig. (**S2**) The stability of Anti-helicobacter pylori IgG seropositivity was demonstrated by forest plot, scatter plot and funnel plot. (Causal effect of Anti-helicobacter pylori IgG seropositivity on PC analysed by MR).

(A) Forest plot of the causal effects of single nucleotide polymorphisms associated with Antibody-Mediated Immune Responses on PC. (B) Leave-one-out of SNPs associated with Anti-helicobacter pylori IgG seropositivity and their risk of PC. Each black point represents result of the IVW MR method applied to estimate the causal effect of Anti-helicobacter pylori IgG seropositivity on PC excluding particular SNP. (C) Scatter plots of genetic associations with Anti-helicobacter pylori IgG seropositivity against the genetic associations with PC. (D) Funnel plot to assess heterogeneity. PC = Pancreatic Cancer, SNP = single nucleotide polymorphism.

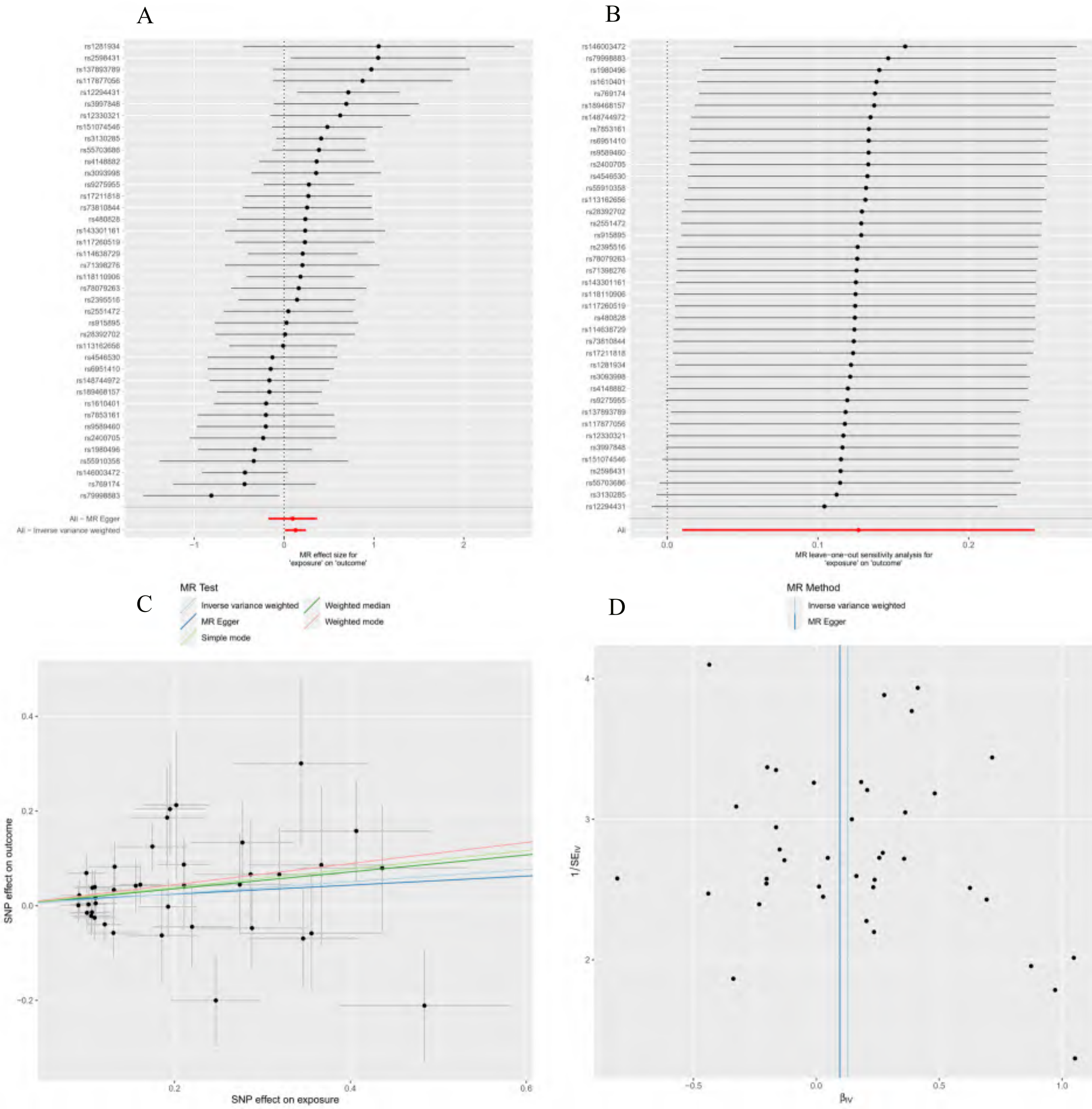

Fig. (**S3**) The stability of Polyomavirus 2 JC VP1 antibody levels was demonstrated by forest plot, scatter plot and funnel plot. (Causal effect of Polyomavirus 2 JC VP1 antibody levels on PC analysed by MR)

(A) Forest plot of the causal effects of single nucleotide polymorphisms associated with Antibody-Mediated Immune Responses on PC. (B) Leave-one-out of SNPs associated with Polyomavirus 2 JC VP1 antibody levels and their risk of PC. Each black point represents result of the IVW MR method applied to estimate the causal effect of Polyomavirus 2 JC VP1 antibody levels on PC excluding particular SNP. (C) Scatter plots of genetic associations with Polyomavirus 2 JC VP1 antibody levels against the genetic associations with PC. (D) Funnel plot to assess heterogeneity. PC = Pancreatic Cancer, SNP = single nucleotide polymorphism.

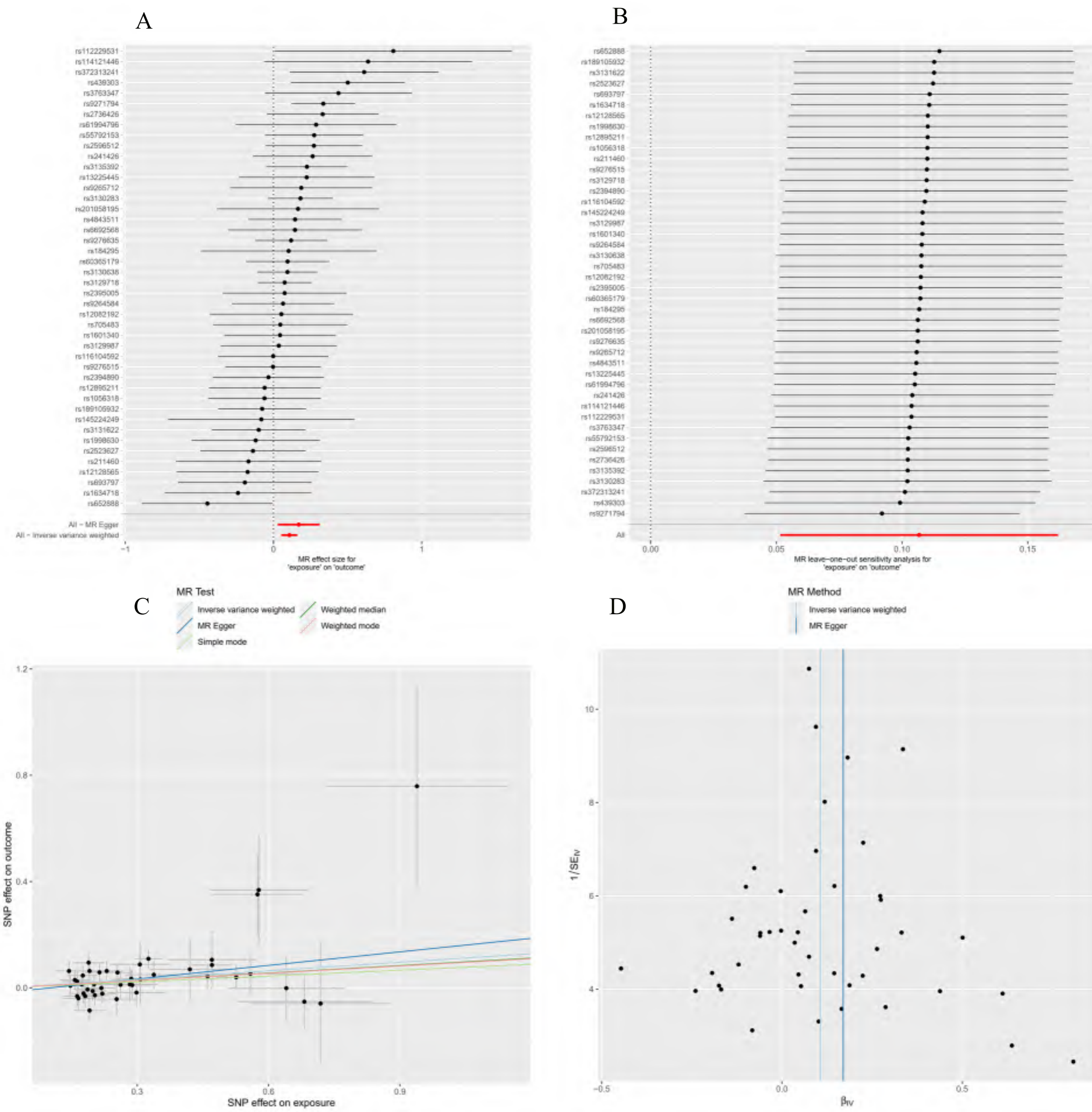

Fig. (S4). The stability of Anti-Merkel cell polyomavirus IgG seropositivity was demonstrated by forest plot, scatter plot and funnel plot. (Causal effect of Anti-Merkel cell polyomavirus IgG seropositivity on PC analysed by MR)

(A) Forest plot of the causal effects of single nucleotide polymorphisms associated with Antibody-Mediated Immune Responses on PC. (B) Leave-one-out of SNPs associated with Anti-Merkel cell polyomavirus IgG seropositivity and their risk of PC. Each black point represents result of the IVW MR method applied to estimate the causal effect of Anti-Merkel cell polyomavirus IgG seropositivity on PC excluding particular SNP. (C) Scatter plots of genetic associations with Anti-Merkel cell polyomavirus IgG seropositivity against the genetic associations with PC. (D) Funnel plot to assess heterogeneity. PC = Pancreatic Cancer, SNP = single nucleotide polymorphism.

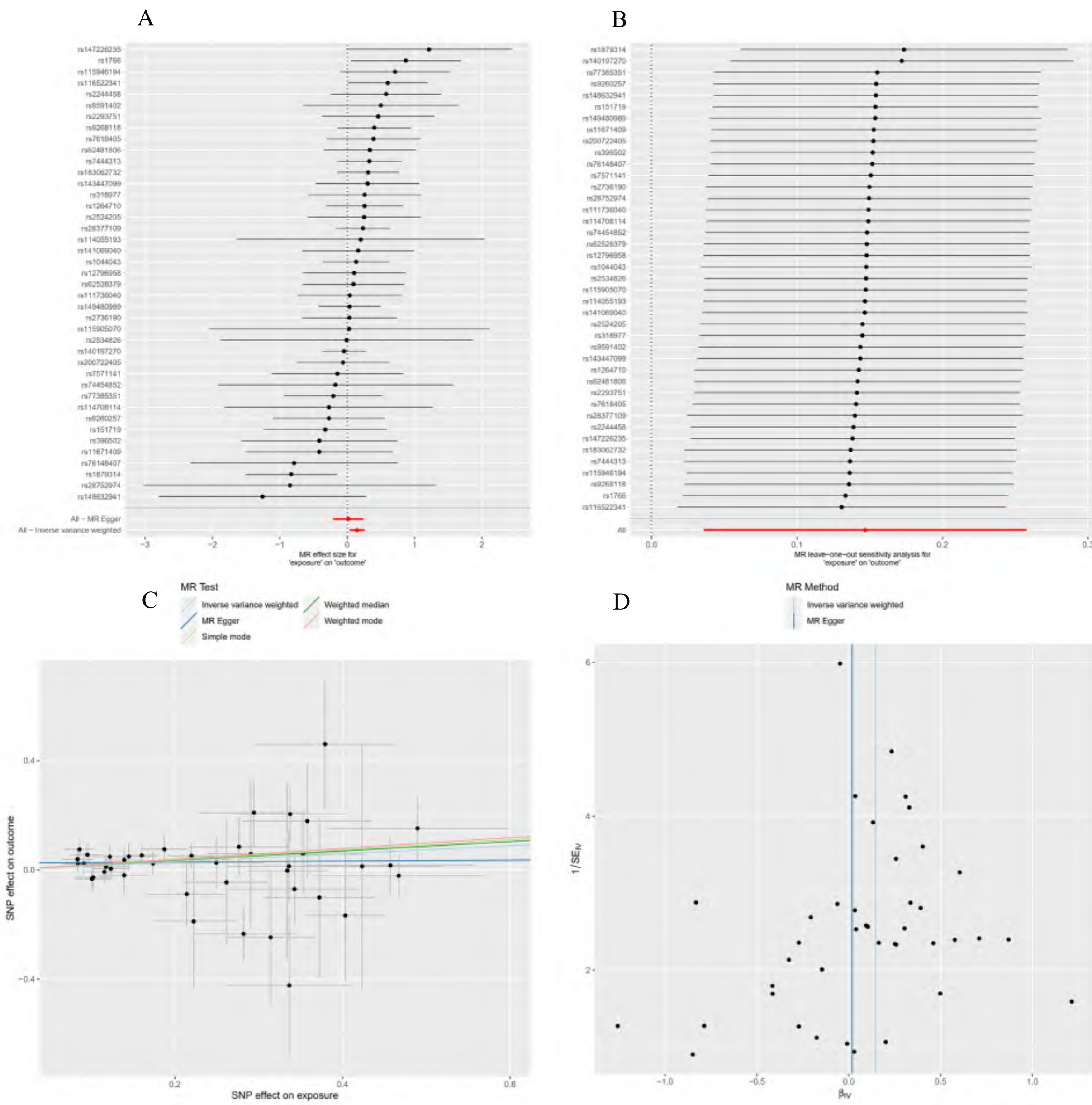

Fig. (S5). The stability of Merkel cell polyomavirus VP1 antibody levels was demonstrated by forest plot, scatter plot and funnel plot. (Causal effect of Merkel cell polyomavirus VP1 antibody levels on PC analysed by MR)

(A) Forest plot of the causal effects of single nucleotide polymorphisms associated with Antibody-Mediated Immune Responses on PC. (B) Leave-one-out of SNPs associated with Merkel cell polyomavirus VP1 antibody levels and their risk of PC. Each black point represents result of the IVW MR method applied to estimate the causal effect of Merkel cell polyomavirus VP1 antibody levels on PC excluding particular SNP. (C) Scatter plots of genetic associations with Merkel cell polyomavirus VP1 antibody levels against the genetic associations with PC. (D) Funnel plot to assess heterogeneity. PC = Pancreatic Cancer, SNP = single nucleotide polymorphism. Abbreviations: IVW , inverse variance weighting ;PC = Pancreatic Cancerr; MR, Mendelian randomization; SNP, single nucleotide polymorphism.
